# The Clinical Society of Maryland

**Published:** 1888-08

**Authors:** 


### THE CLINICAL SOCIETY OF MARYLAND.

STATED MEETING, held May 4, 1888.

The 210th meeting was called to order by the President, Dr. N. G. Keirle.

Dr. Herbert Harlan read the first paper on

### THE TREATMENT OF CORNEAL ULCER WITH ESERINE.

Dr. Samuel Theobald said that he was very much interested in the paper of Dr. Harlan, especially in its bearing on this particular class of cases. He had never used eserine in this connection. Atropia had been the remedy he usually employs, but it was usually tedious in its results. He was glad to know that there was a remedy apparently more efficacious.

Dr. J. T. Wiltshire said that he once treated a case of corneal ulcer with atropia, which, in a short time, produced much pain. Examination showed that the tension was considerably increased.

He then suspected cyclites and decided to use eserine. He did so and it produced marked benefit. The therapeutic action of this drug is interesting in these conditions. It is used to contract the pupils, and how it brings about relief is hard to say. He thinks probably it paralyzes the secretory nerves, prevents their action and thus relieving the tension.

Dr. Hiram Woods said the paper of Dr. Harlan was very clear in treating the subject, and there was little else to be said regarding it. It is an interesting point to find out how eserine acts in this way. We attribute the relief gotten from atropia to the fact that probably a secretory action behind the pupil is lessened after dilatation. But eserine contracts the pupil and this is an opposite condition. Relief here may be due to the action of the drug in promoting drainage from the channels. If there is any danger of iritis or of perforation the use of the drug should be suspended. He had a case last fall where there was a crescentic ulcer present which was on the verge of perforation. He was called away at the time and some one put eserine into the eye. Perforation occurred and an iridectomy had to be performed for relief.

Dr. Samuel Theobald said he thought it would depend a good deal upon the situation of the ulcer in determining its treatment. If it is situated near the margin of the cornea it is best to use eserine. If nearer the centre atropia would be preferable.

Dr. Herbert Harlan, in reply to a question from Dr. Gardner, said no spasm of the ciliary muscles was observed when eserine was used after atropia. He also stated that the number of cases he had reported was greatest in October, possibly because that was the beginning of the oyster season, as twelve cases out of eighteen were caused by oyster shells. In any case where the tension is less than normal it is well to use eserine all the time.

Dr. Hiram Woods said that in some cases an operation to evacuate the anterior chamber and relieve the tension will bring about good results earlier than any remedy. When a crescentic ulcer is about to perforate he would think that this simple operation would be preferable to anything else in accomplishing relief.

Dr. A. K. Bond read a very interesting paper on

### HEGAR'S TEST FOR EARLY PREGNANCY.

Dr. B. B. Browne said that he was very much interested in the paper of Dr. Bond. He is correct in the statement that the rectal examination will reveal a good deal in determining the state of the uterus, but a vaginal examination will give about the same results. It is true that the rectal will enable us to come in contact with the posterior wall of the uterus. Bimanual palpation in some cases will give us all we desire in determining whether or not pregnancy exists. This sign of Hegar adds one other test to the number we already have, some of which are more reliable than that. The softening of early pregnancy is easily recognized. The most reliable means of its detection is the blue discoloration of the cervix and vagina and the test with the thermometer. In the early months of pregnancy the rise of temperature in the cervix is about one degree. Between the cervix and vagina it is about one-half of a degree, In cases where the fœtus is dead it falls about one degree. In regard to the existence of the third sphincter, Dr. Chadwick has recently written a paper in which he fully describes its presence. In cases where tumors and pregnancy exist at the same time this test of Hegar will not be reliable, and also in conditions where the uterus is drawn up high in the pelvis. The blue discoloration and the thermometer are infallible signs.

Dr. Samuel T. Earle said that rectal men had given up the idea of the presence of the third sphincter. It is only an irregular contraction of circular fibres and not a distinct muscle.

Dr. B. B. Browne said that the addition of the posterior ligaments gives a sensation to the touch of a sphincter. He once had a case of a woman, who, while in the third month of pregnancy. was struck in the abdomen and never afterwards was she able to feel the movements of the child. She became alarmed and applied to him to know whether the child was dead or not. The thermometer was introduced into the cervix and the temperature showed a fall of one degree below the normal. The same result was gotten in another similar case. There is no risk in bringing on an abortion unless we penetrate the uterine cavity. These results were reported in the transactions of the Medical and Chirurgical Faculty of Maryland in 1880.

Dr. A. K. Bond said that his reason for writing a paper on this subject was to bring out in English the exact methods practiced by Hegar in determining the existence of pregnancy. So far as American authors are concerned they had failed to do this heretofore. He thinks the test is a valuable one, as it throws another ray of light on the subject of early pregnancy. He had examined several women in the clinic of Dr. Browne and was sure that in some he felt the soft spot referred to.

Dr. R. N. Hall read the next paper on

A CASE OF INTUSSUSCEPTION TREATED WITHOUT OPERATION AND FOLLOWED BY RECOVERY.

Dr. Randolph Winslow said that if the diagnosis of intussusception was the correct one, Dr. Hall ought to have gotten some evidence of sloughed gut. The mortality of this affection is about 70 per cent. Usually there is a discharge of fluid and blood. A tumor is apt to be present, etc. The large mortality in these conditions hardly justifies us in waiting for a spontaneous cure. If the disease is clearly made out it is then time to operate.

Dr. S. T. Earle said he thought from the array of symptoms related by Dr. Hall that he was perfectly justified in making the diagnosis of intussusception.

Dr. L. McLane Tiffany said that in the absence of the sloughed gut the diagnosis of intussusception must remain in doubt. There are a number of conditions that would give rise to these symptoms. The discharge of bloody mucus is one of the few symptoms that will point to a pathological condition, especially so when it occurs in children. He then related two cases of

LAPAROTOMY FOR PURULENT PERITONITIS.

CASE I.—Male, æt. 21 years, barber by occupation, temperate in habits. Was taken suddenly; went to stool, became faint and vomited. He supposed he had colic and procured some medicine for it. This he took, but could not retain it. Feeling ill he went home the next day (Thursday) and sent for his physician, who found, on making an examination, that the patient's belly was very tender and he gave him a purge which acted freely. The next day he felt better, temperature and pulse normal. On Saturday his belly was not swollen and his temperature and pulse still remained normal. Sunday his temperature and pulse were normal, but the abdominal walls were found to be stiff and hard. He had vomited during the night. A large injection was given by the bowels with no effect. Temperature 99°, pulse 90. The suddenness of the onset, the normal pulse and temperature and the stiff condition of the abdomen, led him to make a diagnosis of purulent peritonitis. On Sunday he operated; the intestines were scarlet in appearance; the cæcum was movable, turned upwards and rested against the abdominal walls. This gave rise to obstruction. The incision was made in the median line, and in addition to it a cross incision was also made and the position of the cæcum was restored. The colon then collapsed; the wound was sewed up and dresseed. He died the following day. In this case the inflammation was general; obstruction was due to the turning up of the cæcum. The passage from the bowels on Thursday must have been from the colon.

CASE II.—Now convalescing, male, æt. 19 years. Was taken with discomfort in the right groin as the first symptoms. An injection was given by the bowels and he was put on small doses of morphia and fractional doses of calomel to control the nausea. On Sunday the physical condition was about the same; belly was stiff, Hippocratic countenance present, pulse 45, temperature 99°. The injection was not effective, and was thought to be due to some bowel obstruction. He saw the patient again Thursday, at which time he was lying comfortably on his back. His countenance was brighter and his belly had gone down somewhat, pulse 48. There was noticed a difference in tension over the abdominal walls, being more marked below the umbilicus. He had complained of some pain on the under surface of the penis. A rectal examination showed the presence of something within the pelvis. At this time a diagnosis of purulent peritonitis was made, but he thought it best not to operate at this time, as the pus would probably arise above the pubes, and he could then open the belly and let it out without going into the peritoneal cavity. On Friday the same condition was present. Saturday there was evidence of hardness felt above the pubes. The patient's water was then drawn off. He then opened the belly just above the pubes and let out about one pint of pus; the cavity was washed out and the wound was brought together. By this means he did not get into the peritoneal cavity at all. No fecal matter could anywhere be found; a drainage-tube was put in, and now on the seventh day the temperature and pulse are normal and the tube only goes in 2½ inches, so he is practically well. Obstruction, then, in the first case was due to volvulus of the cæcum, and in the second one it was due to pus. There was no evidence to be found of perforation in the first case.

Dr. J. W. Chambers said that all of the symptoms of acute peritonitis may give rise to those of intestinal obstruction. An intussusception may get well without the gut sloughing. It is hard to account for the trouble in Dr. Tiffany's cases, as nothing could be found to explain the cause. Some foreign body must have gotten into the peritoneum, even though no evidence of it could be found.

Dr. N. G. Keirle said that he had a case where the middle fold of the sigmoid flexure had become fixed, supposed to be the result of peritonitis, as nothing else could be found to explain it.

Dr. S. T. Earle said that he agreed with Dr. Chambers, that some foreign substance must have gotten into the peritoneal cavity to explain the symptoms above referred to.

Dr. L. McLane Tiffany said we must remember that the intestines are very thin and that myriads of organisms infest their track. Whether these organisms penetrate or not is not known. It may be possible that a twist of the bowels may so alter the conditions as to facilitate their activity. In this case no opening of any kind could be found.

Dr. R. M. Hall said that from the history of his case and the symptoms that were found, he thought that he was justified in concluding it one of intussusception. He brought it before the society in order to get the opinion of the members on the subject, but was sorry to say that he had received but little enlightenment on it.

